# Statistical analysis plan for the Pneumatic CompREssion for PreVENting Venous Thromboembolism (PREVENT) trial: a study protocol for a randomized controlled trial

**DOI:** 10.1186/s13063-018-2534-6

**Published:** 2018-03-15

**Authors:** Yaseen Arabi, Fahad Al-Hameed, Karen E. A. Burns, Sangeeta Mehta, Sami Alsolamy, Mohammed Almaani, Yasser Mandourah, Ghaleb A. Almekhlafi, Ali Al Bshabshe, Simon Finfer, Mohammed Alshahrani, Imran Khalid, Yatin Mehta, Atul Gaur, Hassan Hawa, Hergen Buscher, Zia Arshad, Hani Lababidi, Abdulsalam Al Aithan, Jesna Jose, Sheryl Ann I. Abdukahil, Lara Y. Afesh, Maamoun Dbsawy, Abdulaziz Al-Dawood, the PREVENT trial Group, Yaseen Arabi, Abdulaziz Al-Dawood, Sami Alsolamy, Mohamed Hegazy, Maamoun Dbsawy, Sheryl Ann I. Abdukahil, Lara Afesh, Fahad Al-Hameed, Gulam Rasool, Jalal Rifai, Ali S. Mohamed, Ohoud Al Orabi, Yasser Mandourah, Ghaleb Almekhlafi, Dina Al Sufiani, Emad Al Amodi, Mohamed Alkhader, Shatha Awad, Ma. Raylin Cubio Cabal, Jean S. Valerio, Sahar Hassan, Bander Alanazi, Kholoud Alharbi, Ahmad Alenazy, Mohammed Alshahrani, Laila Perlas Asonto, Kathrina Libunao-de Loyola, Ali Al Bshabshe, Abdulmoniem Albahar, Ali Alamri, Abdulsalam Al Aithan, Shahinaz Bashir, Mohammed Almaani, Hani Lababidi, Husain Abdulmuthalib, Pendo Ntinika, Rachelle Pangilinan, Imran Khalid, Abdulaziz Jadkareem, Eman Bawazeer, Sawsan Bassi, Hassan Hawa, Khalid Maghrabi, Mohammad Hijazi, Musaab Abdelhai, Ellen Joy Pagunsan, Marketa Vinklerova, Sangeeta Mehta, Sumesh Shah, Erik Tamberg, Karen Burns, Orla Smith, Marlene Santos, Gyan Sandhu, Jennifer Hodder, Kurtis Salway, Michael Jacka, Lorena McCoshen, Nadia Baig, Atul Gaur, Katrina Ellis, Mary White, Rebecca Gregory, Rob Cameron, Simon Finfer, Anne O’Connor, Elizabeth Yarad, Frances Bass, Naomi Hammond, Hergen Buscher, Claire Reynolds, Karlee McCann, Zia Arshad, Sachin Kumar Srivastava, Avinash Singh, Yatin Mehta, Joby George, Chitra Mehta, Ashish Kumar

**Affiliations:** 1 King Saud Bin Abdulaziz University for Health Sciences, King Abdullah International Medical Research Center, Riyadh, Kingdom of Saudi Arabia; 2 Department of Intensive Care, College of Medicine-Jeddah, King Saud bin Abdulaziz University for Health Sciences, King Abdullah International Medical Research Center, Jeddah, Saudi Arabia; 3 Interdepartmental Division of Critical Care Medicine, St Michael’s Hospital, Li Ka Shing Knowledge Institute, Toronto, ON Canada; 4 Interdepartmental Division of Critical Care Medicine, Mount Sinai Hospital, Toronto, ON Canada; 5 Department of Pulmonary and Critical Care Medicine, King Fahad Medical City, King Saud Bin Abdulaziz University for Health Sciences, Riyadh, Kingdom of Saudi Arabia; 6 Department of Intensive Care Services, Prince Sultan Military Medical City, Riyadh, Saudi Arabia; 7 Department of Critical Care Medicine, King Khalid University, Asir Central Hospital, Abha, Kingdom of Saudi Arabia; 8 Intensive Care, Royal North Shore Hospital and The George Institute for Global Health, University of New South Wales, Sydney, NSW Australia; 9 Department of Emergency and Critical Care, Imam Abdulrahman Bin Faisal University, Al Khobar, Kingdom of Saudi Arabia; 10 Critical Care Section, Department of Medicine, King Faisal Specialist Hospital and Research Center, Jeddah, Kingdom of Saudi Arabia; 11 Institute of Critical Care and Anaesthesiology, Medanta – The Medicity, Gurgaon, Haryana India; 12 Intensive Care Department, Gosford Hospital, Gosford, NSW Australia; 13 Critical Care Medicine Department, King Faisal Specialist Hospital and Research Centre, Riyadh, Kingdom of Saudi Arabia; 14 Intensive Care Medicine, St. Vincent’s Hospital, Sydney, NSW Australia; 15 Department of Anesthesiology and Critical Care, King George’s Medical University, Lucknow, India; 16 Department of Pulmonary and Critical Care Medicine, King Fahad Medical City, Riyadh, Kingdom of Saudi Arabia; 17 Intensive Care and Pulmonary Medicine, King Saud bin Abdulaziz University for Health Sciences, King Abdullah International Medical Research Center, Al Ahsa, Kingdom of Saudi Arabia

**Keywords:** Deep vein thrombosis, Pulmonary embolism, Intermittent pneumatic compression, Adjunct mechanical and pharmacologic DVT prophylaxis, Critically-ill patients

## Abstract

**Background:**

The Pneumatic CompREssion for Preventing VENous Thromboembolism (PREVENT) trial evaluates the effect of adjunctive intermittent pneumatic compression (IPC) with pharmacologic thromboprophylaxis compared to pharmacologic thromboprophylaxis alone on venous thromboembolism (VTE) in critically ill adults.

**Methods/design:**

In this multicenter randomized trial, critically ill patients receiving pharmacologic thromboprophylaxis will be randomized to an IPC or a no IPC (control) group. The primary outcome is “incident” proximal lower-extremity deep vein thrombosis (DVT) within 28 days after randomization. Radiologists interpreting the lower-extremity ultrasonography will be blinded to intervention allocation, whereas the patients and treating team will be unblinded. The trial has 80% power to detect a 3% absolute risk reduction in the rate of proximal DVT from 7% to 4%.

**Discussion:**

Consistent with international guidelines, we have developed a detailed plan to guide the analysis of the PREVENT trial. This plan specifies the statistical methods for the evaluation of primary and secondary outcomes, and defines covariates for adjusted analyses a priori. Application of this statistical analysis plan to the PREVENT trial will facilitate unbiased analyses of clinical data.

**Trial registration:**

ClinicalTrials.gov, ID: NCT02040103. Registered on 3 November 2013;

Current controlled trials, ID: ISRCTN44653506. Registered on 30 October 2013.

**Electronic supplementary material:**

The online version of this article (10.1186/s13063-018-2534-6) contains supplementary material, which is available to authorized users.

## Background

Venous thromboembolism (VTE), including both deep vein thrombosis (DVT) and pulmonary embolism (PE), is a common complication of critical illness and is associated with increased morbidity and mortality [1]. Pharmacologic thromboprophylaxis is recommended for critically ill patients and is supported by high-quality evidence [2]. Despite pharmacologic thromboprophylaxis, 5 to 10% of ICU patients develop DVT [3, 4]. Data regarding the effectiveness of mechanical thromboprophylaxis including intermittent pneumatic compression (IPC) devices and graduated compression stocking (GCS) are scarce. In particular, it is unclear whether the addition of IPC to pharmacologic thromboprophylaxis provides additional protection.

The Pneumatic CompREssion for Preventing VENous Thromboembolism (PREVENT) trial is a concealed, stratified, unblinded, international, multicenter randomized controlled trial (RCT) that examines the effectiveness of adjunct IPC use with pharmacologic thromboprophylaxis compared to pharmacologic thromboprophylaxis (with unfractionated heparin (UFH) or low-molecular-weight heparin (LMWH)) alone on the incidence of proximal lower-extremity DVT in critically ill patients. The trial protocol has been published previously [5].

In this manuscript we describe the PREVENT statistical analysis plan (SAP). The SAP complies with the International Conference on Harmonization of Technical Requirements for Registration of Pharmaceuticals for Human Use, and both the “Statistical principles for clinical trials E9” and “Structure and content of clinical study reports E3” [6, 7]. This SAP identifies the procedures to be applied to the primary and secondary analyses for the entire trial cohort once trial data validation is complete. All analyses were prospectively defined as the SAP was finalized during trial implementation. The SAP was written by the principal investigator and members of the steering committee, who will remain blinded to the study results until all patients have been recruited and the database has been locked. Participant recruitment is expected to be completed by the summer of 2018. The final study report will follow the CONSORT (Consolidated Standards of Reporting Trials) 2010 guidelines for reporting randomized controlled trials [8, 9].

## Methods/design

### Study design

The PREVENT trial will enroll 2000 critically ill patients from 16 hospitals in 4 countries. The study has been approved by the Institutional Review Boards (IRBs) of the participating sites. The trial is registered at ClinicalTrials.gov: (NCT02040103) and Current Controlled Trials (ISRCTN44653506). The study is sponsored by King Abdulaziz City for Science and Technology (Grant number AT 65-34) and King Abdullah International Medical Research Center (Protocol number RC12/045/R), Riyadh, Saudi Arabia. The sponsors have no role in the study design, management or analysis.

**Fig. 1:**
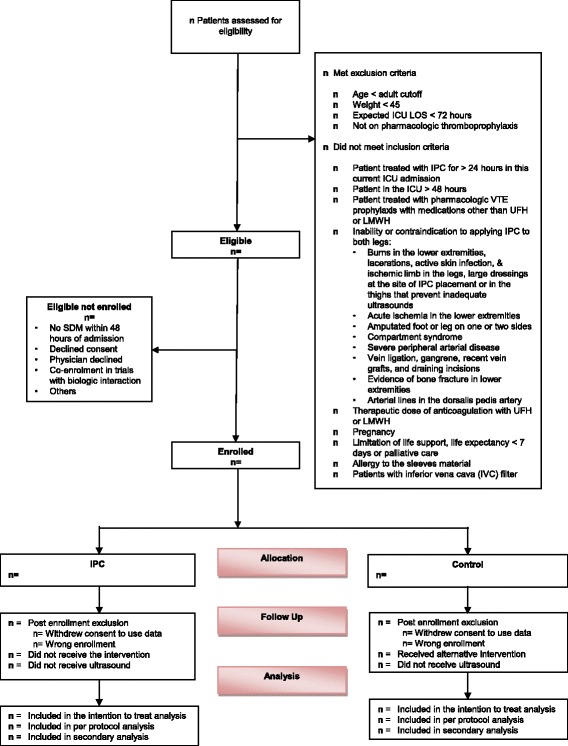
Consolidated Standards of Reporting Trials (CONSORT) flow chart for the PREVENT trial

**Fig. 2:**
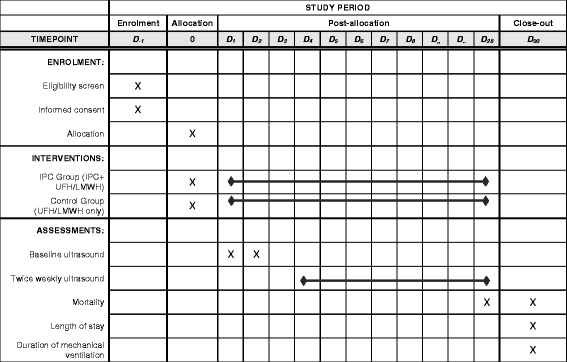
Schedule of enrollment, intervention and assessment for the Pneumatic CompREssion for PreVENting Venous Thromboembolism (PREVENT) trial

All patients will be screened for eligibility within 48 h of ICU admission. Medical-surgical-trauma ICU patients, using accepted age cut-offs in participating adult ICUs (e.g. ≥ 14, 16 or 18 years), who weigh ≥ 45 kg, are expected to stay in ICU for ≥ 72 h and are eligible for pharmacologic thromboprophylaxis with either UFH and LMWH will be enrolled (Fig. 1). Exclusion criteria have been detailed in the protocol manuscript [5]. To enhance the generalizability of our findings, we permit the use of a broad array of IPC devices from various manufacturers intended for DVT prophylaxis. These devices include sequential devices (multi-chamber cuffs) and non-sequential devices (single-chamber cuff); the type of device will be recorded. IPC will be applied continuously for at least 18 h per day. The study intervention will continue for the duration of the ICU stay or up to 28 days after randomization; after which IPC use will be at the discretion of treating team. All enrolled patients will have a bilateral lower-extremity ultrasonography performed by a certified technologist at baseline (within 48 h of enrollment) and twice weekly thereafter until the diagnosis of a lower-extremity DVT or PE, ICU discharge, death, full mobility or 28 days (Fig. 2).

### Study population

A flow chart will be constructed according to the CONSORT guidelines (Fig. 1). We will report the number of patients who were screened, met inclusion or exclusion criteria, and were eligible but not enrolled and reasons for non-enrollment. We will report the number of patients who were randomized to each group, received the allocated intervention, and had at least one ultrasonography performed.

*The Intention-to-treat population* consists of all enrolled patients and will be used for the primary analysis. All enrolled patients will be included regardless of whether or not they received the allocated intervention or had an ultrasonography performed. Post-enrollment exclusion from the intention-to-treat analysis will be restricted to withdrawal of consent to use trial data by the patient or surrogate decision-maker (SDM). However, if the patient or SDM withdraws consent for trial participation but permits collection and use of data, we will include these participants in the planned intention-to-treat analysis. Patients will also be excluded post enrollment if the eligibility criteria were not met. We plan to enroll additional patients to compensate for patients who are excluded post randomization.

*The per-protocol population* consists of all randomized patients who received the allocated intervention *and* had at least one ultrasonography performed. Although the protocol requires that a baseline ultrasonography is performed, enrolled patients may infrequently not have a single trial ultrasonography performed. For example, this circumstance may occur if a patient dies or is transferred out of the participating ICU before an ultrasonography can be performed, if the patient’s ICU stay was shorter than expected (< 72 h) or if there was to difficulty scheduling ultrasonography over weekends.

### Analysis plan

#### Baseline characteristics

We will present baseline characteristics in the two groups of the intention-to-treat cohort (Additional file 1: Online Supplement Table S1). We will compare age, sex, weight, height and Body Mass Index (BMI) between the two study groups. We will compare location immediately prior to ICU admission, Acute Physiology And Chronic Health Evaluation (APACHE) II scores, admission categories, and chronic illnesses as defined by the APACHE II system [10]. We will compare the two groups for the history of heart failure as per New York Heart Association classification and by ejection fraction (if echocardiographic assessment is performed). We will compare pre-defined pre-ICU VTE risk factors, including personal history of VTE, family history of VTE, known thrombophilic states (protein C, protein S or antithrombin deficiency, thrombotic thrombocytopenic purpura, hemolytic-uremic syndrome, activated protein C resistance, factor V Leiden thrombophilia, prothrombin gene mutation, antiphospholipid antibody, hyperhomocysteinemia), post-partum status (within 3 months), estrogen therapy (oral contraceptive or hormone replacement), active malignancy (treatment within the past 6 months or palliation), history of malignancy (within the past 5 years, other than non-melanoma skin cancer), paralysis or immobilization of a lower or upper extremity related to stroke or injury prior to this hospital admission, hospitalization in the past 3 months for any reason (excluding this hospital admission), trauma (including acute spinal cord injury, hip fracture, pelvic fracture, femoral fracture and tibial, fibular, knee or other fractures below knee), recent surgery (in the last 48 h) and acute stroke (in this index hospital admission). We will compare baseline platelet count, international normalized ratio (INR), partial thromboplastin time (PTT), creatinine and partial pressure of arterial oxygen/fraction of inspired oxygen (PaO_2_/FiO_2_) ratio. We will also compare the two study groups for mechanical ventilation, and use of vasopressors, presence of central venous catheters or dialysis catheters in the jugular, subclavian or upper extremities or in the femoral veins. We will compare the number of days from ICU admission and enrollment and use of IPC prior to randomization and duration of use.

### Intervention data

For each group we will report details regarding IPC use and the average daily duration of use (excluding study first and last days because they are usually less than 24 h, Additional file 1: Table S2). We will report reasons for not using IPCs in the IPC group and reasons for IPC use in the control group. We will classify these events as per-protocol and not per-protocol. We will classify IPC devices as sequential or non-sequential and will report the use of thigh- or knee-length sleeves and the use of foot pumps.

### Co-interventions

#### Pharmacologic thromboprophylaxis

We will compare the two study groups for the types of pharmacologic thromboprophylaxis (UFH, LMWH) at the time of enrollment. Because pharmacologic thromboprophylaxis type may be changed by the treating teams, we will report the percentage of patients who received each type of pharmacologic thromboprophylaxis (UFH, LMWH) at any time during the intervention period and for > 50% of the study period.

#### Therapeutic anticoagulation, antiplatelet therapy and anticoagulation for continuous renal replacement therapy

We will compare the two study groups with regard to the number of patients who receive therapeutic anticoagulation (with any agent) for reasons other than VTE during the study period, and the duration of therapeutic anticoagulation. We will report the anticoagulation use for continuous renal replacement therapy (CRRT) (citrate or heparin) during the study period. We will document the use of other anticoagulants during the intervention period (warfarin, other orally administered anticoagulants, danaparoid, argatroban, fondaparinux, lepirudin, others). We will also compare the use of thrombolytic therapy (tissue plasminogen activator (tPA), streptokinase, urokinase) and the use of antiplatelet agents (including aspirin, clopidogrel, ticlopidine, glycoprotein IIb/IIIa inhibitor).

#### Graduated compression stockings

The use of graduated compression stockings (GCS) is not permitted in the trial. If used, we will document the duration and the reasons for use. We will report the number of patients who used GCS in each group and the duration of their use.

#### Central venous catheters, dialysis and arterial catheters

Between groups, we will compare the presence and number of days in situ of central venous catheters or dialysis catheters in the femoral, internal jugular, and subclavian veins, as well as, the presence of a peripherally inserted central catheter (PICC) in the upper extremities. We will also document the presence of arterial lines in the femoral or dorsalis pedis arteries.

#### Other co-interventions

Between groups, we will compare any use of mechanical ventilation, vasopressors, CRRT, intermittent dialysis, or peritoneal dialysis during the intervention period. We will also compare transfusions of packed red blood cells, fresh frozen plasma, platelets and cryoprecipitate between groups. Finally, we will compare the administration of statins, factor VII and vitamin K between treatment groups.

#### Mobility

Because mobility may influence the risk of DVT and because IPC may interfere with mobility, we will compare the highest level of mobility achieved each day and during the intervention duration between treatment groups. Mobility level will be assessed using a pre-defined continuous scale (0 nothing; 1 transfer from bed to chair without standing, 3 sitting in bed/exercises in bed, 4 sitting at edge of bed, 5 standing, 6 transfer from bed to chair with standing, 7 marching in place, 8 walking, and unknown) [11].

#### Diagnostic testing

As this is an open-label study, we will compare the number of diagnostic tests performed for VTE detection between study groups to assess for potential ascertainment bias. Specifically, we will compare the study groups for the number of patients who had undergone at least one ultrasonography of the lower extremities, the time to the first ultrasonography and the number of ultrasonographs of the lower extremities per patient. We will also compare the two groups for the number of patients who underwent ultrasonography of the upper extremities and neck to evaluate for thrombosis, computerized tomography (CT) scan of the chest to evaluate for PE, lung ventilation-perfusion scans, transthoracic and transesophageal echocardiograms (for all indications) and CT scan of the abdomen to evaluate for thrombosis.

### Primary outcome

The primary outcome is incident proximal lower-extremity DVT detected after the third calendar day of enrollment during the intervention period (defined as the number of calendar days from enrollment until the end of the intervention period; that is, diagnosis of lower-extremity DVT, PE, ICU discharge, death, full mobility or 28 days). The primary outcome tests the primary hypothesis that IPC reduces incident proximal lower-extremity DVT (Additional file 1: Table S3).

### Secondary outcomes

A detailed list of secondary outcomes with definitions has already been published and is outlined in Additional file 1: Table S4. These secondary outcomes can be grouped as follows:

1. ***Secondary outcomes related to incident proximal lower-extremity DVT*.** These outcomes test the secondary hypotheses that IPC reduces the extent of incident proximal lower-extremity DVT and reduces central-venous-catheter- and non-central-venous-catheter-related incident proximal lower-extremity DVT

- *Unilateral incident proximal lower-extremity DVT*
- *Bilateral incident proximal lower-extremity DVT*
- *Number of veins with DVT.* As per our protocol, the venous system is examined by documenting compressibility at the following six sites: common femoral, proximal superficial femoral, mid superficial femoral, distal superficial femoral, popliteal veins and trifurcation [5]. We will document the number of veins involved as a measure of DVT extent
- *Complete occlusion (with one vein at least non-compressible)*
- *Incomplete occlusion (with all veins at least partially compressible)*
- *Central-venous-catheter-related incident proximal lower-extremity DVT* as defined previously [5]
- *Non-central-venous-catheter-related incident proximal lower-extremity DVT*

2. ***Secondary outcomes related to lower-extremity DVT other than incident proximal lower-extremity DVT***

- *Prevalent – proximal DVT.* DVT diagnosed on the first ultrasonography within the first three calendar study days are considered “prevalent,” (i.e. reflecting a baseline characteristic); however, some of these DVTs may have occurred after enrollment. While a narrower time window to perform the first ultrasonography would have been ideal, feasibility considerations (i.e. logistics of performing an ultrasonography by a certified technician) mandated a broader time window. Therefore, it is possible that the intervention may reduce prevalent proximal DVT
- *Distal DVT (incident + prevalent).* In general, we will not consider isolated distal lower-extremity DVT. Study surveillance ultrasonography does not include distal calf veins (i.e., peroneal, posterior, anterior tibial, and muscular veins). However, if diagnosed by the treating team, then we will document their occurrence. If lower-extremity DVT occurs in both proximal and distal veins, it will be counted as a proximal DVT
- *All lower-extremity DVT (all proximal and distal)*

3. ***Secondary outcomes related to PE***

- *Incidence of PE*
- *Extent of PE: unilateral, bilateral*
- *PE with cardiopulmonary complications* (supraventricular arrhythmias, ventricular arrhythmias, hypotension (systolic blood pressure ≤ 80 mmHg or decrease of 30 mmHg from baseline) or increase in inotropic support ≥ 50% from pre-event, endotracheal intubation, cardiopulmonary arrest, pulmonary artery hypertension (pulmonary artery (PA) systolic ≥ 60 mmHg or death)
- *Composite all lower-extremity DVT and PE*

4. ***Non-lower-extremity thrombosis***
5. ***Lower-extremity skin pressure ulcers.*** This is a safety outcome, testing whether IPC increases the risk of skin pressure ulcers defined according to the National Pressure Ulcer Advisory Panel (NPUAP) classification
6. ***Lower-extremity ischemia.*** This is a safety outcome, testing whether IPC increases the risk of ischemia in the lower extremities by compromising blood flow
7. ***Serious adverse events (SAEs)*** defined in the study protocol as skin pressure ulcers of categories III and IV or ischemia due to IPC [5]
8. ***Non-tolerance to IPC*** defined as not using IPC for one calendar day or more because of discomfort
9. Mechanical ventilation duration and mechanical-ventilation-free days, vasopressor therapy duration and vasopressor-free days, ICU stay and ICU-free days and hospital length of stay (LOS)
10. ***Mortality outcomes assessed at ICU discharge, 28 days, hospital discharge and 90 days***
11. ***Composite outcome of lower-extremity DVT, PE and 28-day mortality*** **to** address the competing risk of VTE and mortality [12, 13]
12. Serial respiratory, cardiovascular Sequential Organ Failure Assessment (SOFA) scores
13. Serial daily fluid intake, output and balance and vasopressor doses

### Statistical analysis

Categorical variables will be reported as numbers and frequencies, and will be compared using the chi-square test. Continuous variables will be reported as mean and standard deviation or median and interquartile ranges (IQR, Q1–Q3). Continuous variables will be tested using the Student’s *t* test or the Wilcoxon-Mann-Whitney test, as judged appropriate by normality testing. For serial measurements, we will test the change over time and the difference between the two groups over time using a repeated-measures analysis of variance, with no imputation for missing values. For serial measurements, we will use the Bonferroni correction to account for multiple comparisons. Associations will be reported as risk ratios (RR) or hazard ratios (HR) with 95% CIs. Unless otherwise specified, tests will be two-sided and at the 5% significance level. All statistical analyses will be conducted using the SAS software version 9.1.3 or higher (SAS Institute, Cary, NC, USA).

#### Analysis of primary outcome

The primary outcome will be compared in the intention-to-treat and per-protocol cohorts (effectiveness analysis) using chi-square test. Results will be reported as RR with 95% CI (Additional file 1: Table S5). Because some centers may have few events, we will use a generalized linear mixed-model (GLMM) used to estimate adjusted RR after incorporating center/site as random effect [14]. If there is significant difference, we will report relative risk reduction (RRR), absolute risk reduction (ARR) or increase, and number needed to treat (or harm) with 95% CIs. The unadjusted Cox proportional hazard model will also be used to test the null hypothesis and will be used as a secondary analysis tool. We define the hazard function at time *t* as the instantaneous probability of a DVT at time *t* given the patient was free of DVT up to that time. Incident DVT cases will be considered as events. Specifically, we will censor patients who are free of DVT by the end of the 28-day follow-up period, those who die before day 28, and those lost to follow-up (dropouts and lost to follow-up before day 28). We will report hazard ratio (HR) with 95% CI. Kaplan-Meier curves will be generated for the alternative treatment groups and a log-rank test will be used to compare distributions. Although imbalances in baseline characteristics are unlikely with the large sample size, we will conduct an adjusted Cox proportional hazard model with center/site as random effect to adjust for the following factors (defined a priori): enrollment center and type of heparin used (unfractionated vs. low-molecular-weight), in addition to the following variables that are strongly believed to have significant impact on DVT incidence, source of admission to ICU being the ward (compared to the emergency department, operating room and others), trauma, femoral line, dialysis and heart failure.

#### Analysis of secondary outcomes

Secondary outcomes will be compared in the intention-to-treat cohort only using a chi-square test. Results will be reported using RR and 95% CI.

#### Subgroup analyses

The primary outcome will be compared in the intention-to-treat cohort only, in the following a-priori-defined subgroups using a chi-square test (Additional file 1: Table S5). Results will be reported using RR and 95% CI and the multivariable logistic regression will be used to report the results of tests of interactions for these subgroups:

1. UFH and LMWH. Randomization is stratified according to the type of heparin as it may modify the protective effect of IPC on DVT, especially in trauma patients for whom LMWH may be more effective than UFH
2. Femoral CVC at baseline and no femoral CVC at baseline. IPC may have preferential effect in patients with femoral CVC by reducing venous blood stasis. In addition, patients with femoral CVC may have higher baseline rate of DVT, and therefore IPC effect may vary
3. Trauma, postoperative and medical admission diagnoses. IPC may have differential effects on these groups as they have different baseline risks
4. Heart failure and no heart failure (NYHA classification grades III and IV). IPC may have differential effects on these groups as they have different baseline DVT risks
5. Ejection fraction of < 40% or ≥ 40%. This cutoff has been used in several clinical trials to define heart failure with preserved ejection fraction [15–17]. IPC may have differential effects on these groups as they have different baseline DVT risks
6. BMI < 30 and BMI ≥ 30. Given that obesity is a risk factor for DVT, IPC may have differential effects according to BMI
7. Vasopressors versus no vasopressors. Patients on vasopressors are at increased risk for DVT; therefore, IPC may have differential effects according to vasopressor therapy [18]
8. Country: there are differences in patient populations and clinical practices that may influence the baseline DVT risk, including mobility and duration of stay in the ICU. Therefore, IPC may have differential effects according to the country
9. Above-knee sleeves compared to control and below-knee sleeves compared to control
10. Sequential devices compared to control and non-sequential devices compared to control

#### Sensitivity analyses

In a sensitivity analysis, we will consider all lower-extremity DVT and PE events as incident. In another sensitivity analysis, we will consider post-enrollment lower-extremity DVT and PE events if they occur on calendar day 2 or later post enrollment [13]. Because mortality can be a competing outcome with VTE, we plan a sensitivity analysis that is restricted to patients who survive and in the ICU for at least 14 days. Because baseline ultrasonography can be missing, some patients may have a positive non-baseline ultrasonography without a baseline ultrasonography examination, making it difficult to determine whether DVT was a prevelent or an incident event. In these patients, we will have a sensitivity analysis assuming that the missing baseline ultrasonography was negative for DVT and another analysis assuming that the exam was positive. We will examine dose-effect relationship by assessing the relationship of the IPC duration and incident proximal lower-extremity DVT (Table 1 and Additional file 2: SPIRIT 2013 Checklist).

**Table 1:** Summary of analysis plan

Variables	Intention-to-treat cohort	Per Protocol cohort
Baseline characteristics	No statistical comparisons will be performed	None
Intervention and co-interventions	Chi-square, Fisher’s exact test, Mann-Whitney *U* test, *t* test as applicable	None
Primary outcome	1. Primary analysis: chi-square. Report relative risk. Generalized linear mixed model (GLMM) incorporating center/site as random effect. Report as adjusted relative risk. If a significant difference detected: relative risk reduction, absolute risk reduction and number needed to treat (or harm) will be reported 2. Secondary analyses: unadjusted Cox proportional analysis, Kaplan-Meier (KM) curves, adjusted Cox proportional analysis	1. Primary analysis: chi-square and relative risk. Generalized linear mixed model (GLMM) incorporating center/site as random effect. Report as adjusted relative risk. If a significant difference detected: relative risk reduction, absolute risk reduction and number needed to treat (or harm) will be reported. 2. Secondary analyses: unadjusted Cox proportional analysis, KM curves, adjusted Cox proportional analysis
Secondary outcomes	Chi-square. Report relative risk	None
Subgroup analyses	Chi-square. Report relative risk	None

#### Interim analyses

In making a decision to recommend termination of the study, the Data Safety Monitoring Board (DSMB) will be guided by a formal stopping rule based on the primary endpoint of incidence of DVT. The interim test statistics will be the primary outcome analysis for both safety and effectiveness. We will perform two formal interim analyses during the monitoring of the study (when 33% and 67% of the sample size has been achieved). The trial may be stopped for safety (*p* < 0.01) or effectiveness (*p* < 0.001). There will be no plans to terminate the trial for futility. We will account for alpha spending by the O’Brien Fleming method and the final *p* value will be considered at 0.048.

## Discussion

The PREVENT trial examines the effectiveness of adjunct IPC use with pharmacologic thromboprophylaxis compared to pharmacologic thromboprophylaxis (with unfractionated heparin (UFH) or low-molecular-weight heparin (LMWH)) alone on the incidence of proximal lower-extremity DVT in critically ill patients.

### Previous and ongoing studies

The largest trial to date on the effectiveness of IPC is the CLOTS 3 (Clots in Legs Or sTockings after Stroke) trial, which randomized 2876 stroke patients in 94 UK centers to IPC versus no IPC; the use of pharmacologic thromboprophylaxis was at the discretion of the treating team [19]. The primary outcome was proximal vein DVT on screening ultrasonography (performed on legs at 7–10 days of enrollment and, wherever practical, at 25–30 days after enrollment) or any symptomatic DVT in the proximal veins within 30 days of enrollment. The primary outcome occurred in 8.5% patients allocated to IPC and 12.1% of patients allocated to no IPC; with an absolute risk reduction of 3.6% (95% CI 1.4–5.8). Of note, fewer than 25% of patients in CLOTS 3 received pharmacologic thromboprophylaxis. Nevertheless, the protective effect of IPC was observed whether pharmacologic thromboprophylaxis was administered or not (*p* value for interaction 0.897). A recent RCT conducted at the Shanghai Tenth People’s Hospital in China assessed the effectiveness of IPC combined with anticoagulants for the prevention of DVT after total knee arthroplasty [20]. A total of 120 patients were randomized to receive 10 mg of rivaroxaban per day after surgery or to rivaroxaban plus IPC devices. Ultrasonography was performed on postoperative day 9. The primary outcome of DVT occurred in five (8.3%) of 60 patients allocated to the IPC group and 11 (18.3%) of 60 patients allocated to the control group (*p* < 0.01) [20]. The IPCSUPER trial (Intermittent Pneumatic Compression in Surgical Patients at Extremely-high Risk for Venous Thromboembolism) plans to randomize 400 surgical patients in Russia to IPC combined with GCS and pharmacologic thromboprophylaxis (started on the first or second to fifth postoperative day according to the bleeding risk) versus GCS and pharmacologic thromboprophylaxis without IPC [21].

Among the critically ill population, the CIREA2 study by the French critical care group (CRICS) evaluated IPC used with elastic stockings and pharmacologic thromboprophylaxis versus pharmacologic thromboprophylaxis alone on VTE incidence [22]. The study, which planned to enroll 621 patients, has been completed but the results have not been reported. The primary outcome is a composite endpoint (1) non-fatal symptomatic venous thromboembolic event (objectively confirmed) between day 1 and day 6, (2) death due to a PE between day 1 and day 6 and (3) asymptomatic DVT detected by ultrasonography systematically performed at day 6. This study differs from the PREVENT trial by including elastic stockings in addition to IPC. Also, the ultrasonography assessment was only done once (on day 6) and without baseline ultrasonography assessment.

Detailed description of the strengths and limitations of the PREVENT trial have been published previously. In this SAP, we outline details of the planned analyses in advance of trial completion.

The PREVENT trial evaluates whether IPC, in addition to pharmacologic thromboprophylaxis, compared with pharmacologic thromboprophylaxis alone reduce DVTs in critically ill adults. The PREVENT study is expected to provide evidence that will inform practice regarding the best thromboprophylaxis for critically ill adult patients and contribute to future clinical practice guidelines and patient-safety initiatives.

### Trial status

The first patient was enrolled in July 2014. As of January 2018, a total of 1620 patients have been enrolled from 16 centers in Saudi Arabia, Canada, Australia and India. We expect to complete recruitment of 2000 patients by the summer of 2018.

## Additional files

Additional file 1: Online supplement. (DOCX 63 kb)

Additional file 2: SPIRIT 2013 Checklist. (PDF 238 kb)

## Abbreviations

CI: Confidence interval
DVT: Deep vein thrombosis
GCS: Graduated compression stocking
HR: Hazard ratio
ICU: Intensive care unit
INR: International normalized ratio
IPC: Intermittent pneumatic compression
IQR: Interquartile range
IU: International units
LMWH: Low-molecular-weight heparin
LOS: Length of stay
PE: Pulmonary embolism
RCT: Randomized controlled trial
RR: Relative risk
RRR: Relative risk reduction
SAP: Statistical analysis plan
UFH: Unfractionated heparin
VTE: Venous thromboembolism
