# Supplementary material for: Statistical analysis plan for the Pneumatic CompREssion for PreVENting Venous Thromboembolism (PREVENT) trial: a study protocol for a randomized controlled trial
Source: Trials. 2018 Mar 15;19:182. doi: 10.1186/s13063-018-2534-6 (PMC5856363; doi:10.1186/s13063-018-2534-6)
Supplement: Supplementary file 1 — Online supplement. (DOCX 63 kb) [file 13063_2018_2534_MOESM1_ESM.docx]

**Additional file 1**

| **Table S1:** Baseline characteristics – ITT Population. |  |  |
| --- | --- | --- |
| **Characteristic** | **IPC Group (N=XXXX)** | **Control Group (N=XXXX)** |
| Age (Years) - Mean (SD) | xx (xx.x) | xx (xx.x) |
| Male sex - n (%) | xxxx/xxxx (xx.x) | xxxx/xxxx (xx.x) |
| Height (cm) - Mean (SD) | xx (xx.x) | xx (xx.x) |
| Weight (kg) - Mean (SD) | xx (xx.x) | xx (xx.x) |
| BMI (kg/m^2^) - Mean (SD) | xx (xx.x) | xx (xx.x) |
|  |  |  |
| Location prior to ICU admission - n (%) |  |  |
| Emergency room | xxxx (xx.x) | xxxx (xx.x) |
| Hospital ward | xxxx (xx.x) | xxxx (xx.x) |
| Operating room | xxxx (xx.x) | xxxx (xx.x) |
| Other hospital (ICU or ward) | xxxx (xx.x) | xxxx (xx.x) |
| Other | xxxx (xx.x) | xxxx (xx.x) |
|  |  |  |
| APACHE II- Mean (SD) | xx (xx.x) | xx (xx.x) |
| Diagnostic categories (as defined by APACHE II system)-n (%) |  |  |
| Trauma (Non-Operative Trauma and Post- Operative Trauma) | xxxx (xx.x) | xxxx (xx.x) |
| Medical (Non-Operative Non-Trauma) | xxxx (xx.x) | xxxx (xx.x) |
| Post-Operative Non-Trauma | xxxx (xx.x) | xxxx (xx.x) |
|  |  |  |
| Chronic Health Illnesses - n (%) |  |  |
| None | xxxx (xx.x) | xxxx (xx.x) |
| Liver disease | xxxx (xx.x) | xxxx (xx.x) |
| Cardiovascular disease | xxxx (xx.x) | xxxx (xx.x) |
| Respiratory disease | xxxx (xx.x) | xxxx (xx.x) |
| Renal disease | xxxx (xx.x) | xxxx (xx.x) |
| Immunosuppression | xxxx (xx.x) | xxxx (xx.x) |
|  |  |  |
| Patients with heart failure - n (%) | xxxx (xx.x) | xxxx (xx.x) |
| NY Class I | xxxx (xx.x) | xxxx (xx.x) |
| NY Class II | xxxx (xx.x) | xxxx (xx.x) |
| NY Class III | xxxx (xx.x) | xxxx (xx.x) |
| NY Class IV | xxxx (xx.x) | xxxx (xx.x) |
|  |  |  |
| Ejection fraction |  |  |
| Not tested | xxxx (xx.x) | xxxx (xx.x) |
| >=40% | xxxx (xx.x) | xxxx (xx.x) |
| <40% | xxxx (xx.x) | xxxx (xx.x) |
|  |  |  |
| Pre-ICU conditions that may influence VTE risk - n (%) |  |  |
| Personal history of VTE | xxxx (xx.x) | xxxx (xx.x) |
| Family history of VTE | xxxx (xx.x) | xxxx (xx.x) |
| Known thrombophilic state | xxxx (xx.x) | xxxx (xx.x) |
| Post-partum (within 3 months) | xxxx (xx.x) | xxxx (xx.x) |
| Estrogen therapy | xxxx (xx.x) | xxxx (xx.x) |
| Active malignancy (treatment within past 6 months or palliation) | xxxx (xx.x) | xxxx (xx.x) |
| History of malignancy (past 5 years; other than non-melanoma skin cancer) | xxxx (xx.x) | xxxx (xx.x) |
| Paralysis or immobilization of a lower or upper extremity related to stroke or injury  prior to this hospital admission | xxxx (xx.x) | xxxx (xx.x) |
| Hospitalization in the past 3 months for any reason (excluding this hospital admission) | xxxx (xx.x) | xxxx (xx.x) |
| Trauma | xxxx (xx.x) | xxxx (xx.x) |
| Recent surgery (in the last 48 hours) | xxxx (xx.x) | xxxx (xx.x) |
| Acute stroke (this hospital admission) | xxxx (xx.x) | xxxx (xx.x) |
| Others | xxxx (xx.x) | xxxx (xx.x) |
| None | xxxx (xx.x) | xxxx (xx.x) |
|  |  |  |
| Laboratory results prior to randomization - Mean (SD) |  |  |
| INR (highest) | xx (xx.x) | xx (xx.x) |
| Creatinine (µmol/L) | xx (xx.x) | xx (xx.x) |
| Platelets (10^9^/L) | xx (xx.x) | xx (xx.x) |
| PTT (highest) | xx (xx.x) | xx (xx.x) |
| Hemoglobin (g/L) | xx (xx.x) | xx (xx.x) |
|  |  |  |
| Mechanical ventilation on Day 1 - n (%) | xxxx (xx.x) | xxxx (xx.x) |
| Vasopressor use on Day 1 - n (%) | xxxx (xx.x) | xxxx (xx.x) |
|  |  |  |
| Central Venous Lines on Day 1 |  |  |
| Jugular, subclavian or upper extremities- n (%) | xxxx (xx.x) | xxxx (xx.x) |
| Femoral- n (%) | xxxx (xx.x) | xxxx (xx.x) |
| None- n (%) | xxxx (xx.x) | xxxx (xx.x) |
|  |  |  |
| Number of days from ICU admission to randomization -Median (Q1,Q3) | xx (xx, xx) | xx (xx, xx) |
| Number of hours on IPC from ICU admission to the time of randomization -Median (Q1,Q3) | xx (xx, xx) | xx (xx, xx) |

Denominator of the percentage is the total number of subjects in each group in the ITT population.

| **Table S2:** Summary of interventions and co-interventions in the intention-to-treat population. Calculations are provided for the all patients in each group. | | |
| --- | --- | --- |
| **Variable** | **IPC Group (N=XXXX)** | **Control Group (N=XXXX)** |
| Use of IPC |  |  |
| Number of patients receiving IPC during the study period | xxxx (xx.x) | xxxx (xx.x) |
| Average daily no. of hours of IPC on both legs during study period- Median (Q1, Q3) | xx (xx, xx) | xx (xx, xx) |
|  |  |  |
| Device type |  |  |
| Sequential | xxxx (xx.x) | xxxx (xx.x) |
| Non-sequential | xxxx (xx.x) | xxxx (xx.x) |
|  |  |  |
| Sleeves |  |  |
| Below knee sleeves | xxxx (xx.x) | xxxx (xx.x) |
| Above knee sleeves | xxxx (xx.x) | xxxx (xx.x) |
|  |  |  |
| Number of patients on foot pump - n(%) | xxxx (xx.x) | xxxx (xx.x) |
|  |  |  |
| Total number of sleeves used during intervention period- Median (Q1, Q3) | xx (xx, xx) | xx (xx, xx) |
|  |  |  |
| Co-Interventions |  |  |
| Pharmacologic prophylaxis at the time of enrollment |  |  |
| Prophylactic UFH | xxxx (xx.x) | xxxx (xx.x) |
| Prophylactic LMWH | xxxx (xx.x) | xxxx (xx.x) |
|  |  |  |
| Pharmacologic prophylaxis at any time during intervention period - n (%) |  |  |
| Prophylactic UFH | xxxx (xx.x) | xxxx (xx.x) |
| Prophylactic LMWH | xxxx (xx.x) | xxxx (xx.x) |
| Pharmacologic prophylaxis for >50% of the intervention period - n (%) |  |  |
| Prophylactic UFH | xxxx (xx.x) | xxxx (xx.x) |
| Prophylactic LMWH | xxxx (xx.x) | xxxx (xx.x) |
|  |  |  |
| Therapeutic anticoagulation for reasons other than VTE | xxxx (xx.x) | xxxx (xx.x) |
| Duration of therapeutic anticoagulation for reasons other than VTE– Median (Q1, Q3) | xx (xx, xx) | xx (xx, xx) |
|  |  |  |
| Anticoagulation for CRRT- Citrate | xxxx (xx.x) | xxxx (xx.x) |
| Anticoagulation for CRRT- Low dose Heparin | xxxx (xx.x) | xxxx (xx.x) |
|  |  |  |
| Other Anticoagulation |  |  |
| Warfarin | xxxx (xx.x) | xxxx (xx.x) |
| Other oral anticoagulants | xxxx (xx.x) | xxxx (xx.x) |
| Danoparoid | xxxx (xx.x) | xxxx (xx.x) |
| Argatroban | xxxx (xx.x) | xxxx (xx.x) |
| Fondaparinux | xxxx (xx.x) | xxxx (xx.x) |
| Lepirudin | xxxx (xx.x) | xxxx (xx.x) |
| Thrombolytics (TpA / Streptokinase / Urokinase) | xxxx (xx.x) | xxxx (xx.x) |
|  |  |  |
| Anti-platelet Therapy |  |  |
| Aspirin | xxxx (xx.x) | xxxx (xx.x) |
| Plavix | xxxx (xx.x) | xxxx (xx.x) |
| Glycoprotein IIa / IIIb Inhibitor | xxxx (xx.x) | xxxx (xx.x) |
| Ticlopidine | xxxx (xx.x) | xxxx (xx.x) |
|  |  |  |
| GCS used - n(%) | xxxx (xx.x) | xxxx (xx.x) |
| Duration of GCS use – Median (Q1,Q3) | xx (xx, xx) | xx (xx, xx) |
|  |  |  |
| Central Venous Lines during intervention period - n (%) |  |  |
| Femoral- n (%) | xxxx (xx.x) | xxxx (xx.x) |
| Jugular or subclavian | xxxx (xx.x) | xxxx (xx.x) |
| Upper extremities PICC- n (%) | xxxx (xx.x) | xxxx (xx.x) |
| None- n (%) | xxxx (xx.x) | xxxx (xx.x) |
|  |  |  |
| Arterial line in lower extremities during intervention period - n (%) |  |  |
| Femoral | xxxx (xx.x) | xxxx (xx.x) |
| Dorsalis pedis | xxxx (xx.x) | xxxx (xx.x) |
|  |  |  |
| Mechanical ventilation during intervention period - n (%) | xxxx (xx.x) | xxxx (xx.x) |
| Vasopressor use during intervention period - n (%) | xxxx (xx.x) | xxxx (xx.x) |
| Renal replacement therapy |  |  |
| Continuous Renal Replacement Therapy | xxxx (xx.x) | xxxx (xx.x) |
| Intermittent dialysis | xxxx (xx.x) | xxxx (xx.x) |
| Peritoneal dialysis | xxxx (xx.x) | xxxx (xx.x) |
|  |  |  |
| Transfusion |  |  |
| PRBC transfusion | xxxx (xx.x) | xxxx (xx.x) |
| FFP | xxxx (xx.x) | xxxx (xx.x) |
| Cryoprecipitate | xxxx (xx.x) | xxxx (xx.x) |
| Platelets | xxxx (xx.x) | xxxx (xx.x) |
|  |  |  |
| Other medications |  |  |
| Statins | xxxx (xx.x) | xxxx (xx.x) |
| Factor VII | xxxx (xx.x) | xxxx (xx.x) |
| Vitamin K | xxxx (xx.x) | xxxx (xx.x) |
|  |  |  |
| Mobility (highest level of during intervention period) |  |  |
| 0- Nothing | xxxx (xx.x) | xxxx (xx.x) |
| 1- Transfer to bed to chair without standing | xxxx (xx.x) | xxxx (xx.x) |
| 2- Sitting in bed/exercises in bed | xxxx (xx.x) | xxxx (xx.x) |
| 3- Sitting at edge of bed | xxxx (xx.x) | xxxx (xx.x) |
| 4- Standing | xxxx (xx.x) | xxxx (xx.x) |
| 5- Transfer from bed to chair with standing | xxxx (xx.x) | xxxx (xx.x) |
| 6- Marching in place | xxxx (xx.x) | xxxx (xx.x) |
| 7- Walking | xxxx (xx.x) | xxxx (xx.x) |
| 8- Unknown | xxxx (xx.x) | xxxx (xx.x) |
| IVC filter placed for prophylaxis | xxxx (xx.x) | xxxx (xx.x) |
|  |  |  |
| Diagnostic Testing, n (%) |  |  |
| Lower extremities ultrasonography |  |  |
| Patients with at least one ultrasonography – n (%) | xxxx (xx.x) | xxxx (xx.x) |
| Days to first ultrasonography – Median (Q1,Q3) | xx (xx, xx) | xx (xx, xx) |
| Number of ultrasonography – Median (Q1,Q3) | xx (xx, xx) | xx (xx, xx) |
|  |  |  |
| Ultrasonography for upper extremities and neck to evaluate for thrombosis – n (%) | xxxx (xx.x) | xxxx (xx.x) |
| Patients with spiral CT (also called CT angiograms or helical CTscan) of chest to evaluate for PE– n (%) | xxxx (xx.x) | xxxx (xx.x) |
| Patients with V/Q scan of the lungs– n (%) | xxxx (xx.x) | xxxx (xx.x) |
| Patients with CT scan of the abdomen to evaluate thrombosis– n (%) | xxxx (xx.x) | xxxx (xx.x) |
| Patients with transthoracic echocardiograms– n (%) | xxxx (xx.x) | xxxx (xx.x) |
| Patients with transesophageal echograms– n (%) | xxxx (xx.x) | xxxx (xx.x) |

**IPC**: Intermittent Pneumatic Compression; **UFH**: Unfractionated Heparin; **LMWH**: Low Molecular Weight Heparin; **PRCB**: Packed Red Blood Cells; **CRRT**: Continuous Renal Replacement Ther

**Table S3:** Primary Outcome: Incident proximal deep venous thrombosis (DVT) in the lower extremities

|  | **ITT Population** | | | **PP Population** | | |
| --- | --- | --- | --- | --- | --- | --- |
| **Variable** | **IPC Group**  **(N=XXXX)** | **Control Group**  **(N=XXXX)** | **P-value** | **IPC Group**  **(N=XXXX)** | **Control Group**  **(N=XXXX)** | **P-value** |
| Incident – Proximal lower extremities DVT - n/N (%) | xx/xxxx (xx.x) | xx/xxxx (xx.x) | x.xxx | xx/xxxx (xx.x) | xx/xxxx (xx.x) | x.xxx |
| Relative Risk, (95% CI) | xx.x (xx.x, xx.x) | |  | xx.x (xx.x, xx.x) | |  |
| Adjusted Relative Risk*, (95% CI) | xx.x (xx.x, xx.x) | |  | xx.x (xx.x, xx.x) | |  |
| Days to event - Median (Q1,Q3) | xx (xx, xx) | xx (xx, xx) |  | xx (xx, xx) | xx (xx, xx) |  |
| Unadjusted hazard Ratio (95% CI) | x.xx (x.xx, x.xx) | | x.xxx | x.xx (x.xx, x.xx) | | x.xxx |
| Adjusted Hazard Ratio# (95% CI) | x.xx (x.xx, x.xx) | | x.xxx | x.xx (x.xx, x.xx) | | x.xxx |

*The GLMM will be used to estimate adjusted relative risk after incorporating center as random effect.

#The cox regression will be used to estimate adjusted hazard ratio after incorporating center as random effect.

**Table S4:** Secondary Outcomes ITT Population

| **Variable** | **IPC Group**  **(N=XXXX)** | **Control Group**  **(N=XXXX)** | **Relative Risk, (95% CI)** | **P-value** |
| --- | --- | --- | --- | --- |
| **Features of Incident – Proximal lower extremities DVT** |  |  |  |  |
| Unilateral DVT – n (%) | xxxx (xx.x) | xxxx (xx.x) | x.xx (x.xx , x.xx) | x.xxx |
| Bilateral DVT – n (%) | xxxx (xx.x) | xxxx (xx.x) | x.xx (x.xx , x.xx) | x.xxx |
| Number of veins with DVT - Median (Q1,Q3) | xx (xx, xx) | xx (xx, xx) |  | x.xxx |
| Complete occlusion (with one vein at least non- compressible) | xxxx (xx.x) | xxxx (xx.x) | x.xx (x.xx , x.xx) | x.xxx |
| Incomplete occlusion (with all veins at least partially compressible) | xxxx (xx.x) | xxxx (xx.x) | x.xx (x.xx , x.xx) | x.xxx |
|  |  |  |  |  |
| Central venous catheter –related DVT |  |  |  |  |
| Yes | xxxx (xx.x) | xxxx (xx.x) | x.xx (x.xx , x.xx) | x.xxx |
| No | xxxx (xx.x) | xxxx (xx.x) | x.xx (x.xx , x.xx) | x.xxx |
| Prevalent – proximal DVT- n (%) | xxxx (xx.x) | xxxx (xx.x) | x.xx (x.xx , x.xx) | x.xxx |
| Distal DVT (Incident + Prevalent) - n/N (%) | xxxx (xx.x) | xxxx (xx.x) | x.xx (x.xx , x.xx) | x.xxx |
| All lower extremity DVT (all proximal and distal) - n/N (%) | xxxx (xx.x) | xxxx (xx.x) | x.xx (x.xx , x.xx) | x.xxx |
|  |  |  |  |  |
| Pulmonary Embolism– n/N (%) | xxxx (xx.x) | xxxx (xx.x) | x.xx (x.xx , x.xx) | x.xxx |
| Extent of PE |  |  |  |  |
| Unilateral – n (%) | xxxx (xx.x) | xxxx (xx.x) | x.xx (x.xx , x.xx) | x.xxx |
| Bilateral – n (%) | xxxx (xx.x) | xxxx (xx.x) | x.xx (x.xx , x.xx) | x.xxx |
| PE with cardiopulmonary complications | xxxx (xx.x) | xxxx (xx.x) | x.xx (x.xx , x.xx) | x.xxx |
| Composite all lower extremity DVT and PE | xxxx (xx.x) | xxxx (xx.x) | x.xx (x.xx , x.xx) | x.xxx |
|  |  |  |  |  |
| **Non lower extremities thrombosis** |  |  |  |  |
| Upper extremity or jugular– n (%) | xxxx (xx.x) | xxxx (xx.x) | x.xx (x.xx , x.xx) | x.xxx |
| Mesenteric vein – n (%) | xxxx (xx.x) | xxxx (xx.x) | x.xx (x.xx , x.xx) | x.xxx |
| Vena Cava – n (%) | xxxx (xx.x) | xxxx (xx.x) | x.xx (x.xx , x.xx) | x.xxx |
| Other (including portal, hepatic, splenic, – n (%) | xxxx (xx.x) | xxxx (xx.x) | x.xx (x.xx , x.xx) | x.xxx |
|  |  |  |  |  |
| **Lower extremity skin ulceration** (Highest stage during intervention period) |  |  |  |  |
| Stage I: Non-blanchable erythema | xxxx (xx.x) | xxxx (xx.x) | x.xx (x.xx , x.xx) | x.xxx |
| Stage II: Partial thickness | xxxx (xx.x) | xxxx (xx.x) | x.xx (x.xx , x.xx) | x.xxx |
| Stage III: Full thickness skin loss | xxxx (xx.x) | xxxx (xx.x) | x.xx (x.xx , x.xx) | x.xxx |
| Stage IV: Full thickness tissue loss | xxxx (xx.x) | xxxx (xx.x) | x.xx (x.xx , x.xx) | x.xxx |
|  |  |  |  |  |
| **Leg-Ischemia**(Highest during intervention period) |  |  |  |  |
| Toes | xxxx (xx.x) | xxxx (xx.x) | x.xx (x.xx , x.xx) | x.xxx |
| Up to foot | xxxx (xx.x) | xxxx (xx.x) | x.xx (x.xx , x.xx) | x.xxx |
| Up to leg | xxxx (xx.x) | xxxx (xx.x) | x.xx (x.xx , x.xx) | x.xxx |
| Up to thigh | xxxx (xx.x) | xxxx (xx.x) | x.xx (x.xx , x.xx) | x.xxx |
|  |  |  |  |  |
| Serious adverse events (SAEs) | xxxx (xx.x) | xxxx (xx.x) | x.xx (x.xx , x.xx) | x.xxx |
|  |  |  |  |  |
| Duration of MV - Median (Q1,Q3) | xx (xx, xx) | xx (xx, xx) |  | x.xxx |
| Mechanical Ventilation free days - Median (Q1,Q3) | xx (xx, xx) | xx (xx, xx) |  | x.xxx |
| Duration of Vasopressor use - Median (Q1,Q3) | xx (xx, xx) | xx (xx, xx) |  | x.xxx |
| Vasopressor free days - Median (Q1,Q3) | xx (xx, xx) | xx (xx, xx) |  | x.xxx |
| ICU LOS - Median (Q1,Q3) | xx (xx, xx) | xx (xx, xx) |  | x.xxx |
| ICU-free days | xx (xx, xx) | xx (xx, xx) |  | x.xxx |
| Hospital LOS - Median (Q1,Q3) | xx (xx, xx) | xx (xx, xx) |  | x.xxx |
|  |  |  |  |  |
| ICU mortality – n (%) | xxxx (xx.x) | xxxx (xx.x) | x.xx (x.xx , x.xx) | x.xxx |
| 28-day mortality | xxxx (xx.x) | xxxx (xx.x) | x.xx (x.xx , x.xx) | x.xxx |
| Composite endpoint of lower extremities DVT, PE and 28-day mortality | xxxx (xx.x) | xxxx (xx.x) | x.xx (x.xx , x.xx) | x.xxx |
| Hospital Mortality – n (%) | xxxx (xx.x) | xxxx (xx.x) | x.xx (x.xx , x.xx) | x.xxx |
| 90-day Mortality – n (%) | xxxx (xx.x) | xxxx (xx.x) | x.xx (x.xx , x.xx) | x.xxx |

Denominator of the percentage is the total number of subjects in each group in the ITT and PP population.

Mechanical Ventilation free days Vasopressor free days and ICU free days are calculated based on 28-d observation

**DVT**: Deep Vein Thrombosis; **PE**: Pulmonary Embolism; **LOS**: Length of Stay

**Table S5:** Subgroup analyses

|  | **Incident –Proximal DVT** | | | | |
| --- | --- | --- | --- | --- | --- |
|  | **IPC Group**  **(N=XXXX)** | **Control Group**  **(N=XXXX)** | **RR (95% CI)** | **P-value** | **P-value for interaction** |
| UFH | xxxx/xxxx (xx.x) | xxxx/xxxx (xx.x) | x.xx (x.xx, x.xx) | x.xxx | x.xxx |
| LMWH | xxxx/xxxx (xx.x) | xxxx/xxxx (xx.x) | x.xx (x.xx, x.xx) | x.xxx |  |
|  |  |  |  |  |  |
| Femoral CVC at baseline | xxxx/xxxx (xx.x) | xxxx/xxxx (xx.x) | x.xx (x.xx, x.xx) | x.xxx | x.xxx |
| No Femoral CVC at baseline | xxxx/xxxx (xx.x) | xxxx/xxxx (xx.x) | x.xx (x.xx, x.xx) | x.xxx |  |
|  |  |  |  |  |  |
| Trauma | xxxx/xxxx (xx.x) | xxxx/xxxx (xx.x) | x.xx (x.xx, x.xx) | x.xxx | x.xxx |
| Post-operative | xxxx/xxxx (xx.x) | xxxx/xxxx (xx.x) | x.xx (x.xx, x.xx) | x.xxx |  |
| Medical | xxxx/xxxx (xx.x) | xxxx/xxxx (xx.x) | x.xx (x.xx, x.xx) | x.xxx |  |
|  |  |  |  |  |  |
| Heart failure | xxxx/xxxx (xx.x) | xxxx/xxxx (xx.x) | x.xx (x.xx, x.xx) | x.xxx | x.xxx |
| No heart failure | xxxx/xxxx (xx.x) | xxxx/xxxx (xx.x) | x.xx (x.xx, x.xx) | x.xxx |  |
|  |  |  |  |  |  |
| Ejection fraction of ≥40% | xxxx/xxxx (xx.x) | xxxx/xxxx (xx.x) | x.xx (x.xx, x.xx) | x.xxx | x.xxx |
| Ejection fraction of <40% | xxxx/xxxx (xx.x) | xxxx/xxxx (xx.x) | x.xx (x.xx, x.xx) | x.xxx |  |
|  |  |  |  |  |  |
| BMI <30 | xxxx/xxxx (xx.x) | xxxx/xxxx (xx.x) | x.xx (x.xx, x.xx) | x.xxx | x.xxx |
| BMI >30 | xxxx/xxxx (xx.x) | xxxx/xxxx (xx.x) | x.xx (x.xx, x.xx) | x.xxx |  |
|  |  |  |  |  |  |
| Vasopressors | xxxx/xxxx (xx.x) | xxxx/xxxx (xx.x) | x.xx (x.xx, x.xx) | x.xxx | x.xxx |
| No vasopressors | xxxx/xxxx (xx.x) | xxxx/xxxx (xx.x) | x.xx (x.xx, x.xx) | x.xxx |  |
|  |  |  |  |  |  |
| Saudi Arabia | xxxx/xxxx (xx.x) | xxxx/xxxx (xx.x) | x.xx (x.xx, x.xx) | x.xxx | x.xxx |
| Canada | xxxx/xxxx (xx.x) | xxxx/xxxx (xx.x) | x.xx (x.xx, x.xx) | x.xxx |  |
| Australia | xxxx/xxxx (xx.x) | xxxx/xxxx (xx.x) | x.xx (x.xx, x.xx) | x.xxx |  |
| India | xxxx/xxxx (xx.x) | xxxx/xxxx (xx.x) | x.xx (x.xx, x.xx) | x.xxx |  |
|  |  |  |  |  |  |
| Above knee sleeves compared to control* | xxxx/xxxx (xx.x) | xxxx/xxxx (xx.x) | x.xx (x.xx, x.xx) | x.xxx | x.xxx |
| Below knee sleeves compared to control* | xxxx/xxxx (xx.x) | xxxx/xxxx (xx.x) | x.xx (x.xx, x.xx) | x.xxx |  |
|  |  |  |  |  |  |
| Sequential devises compared to control** | xxxx/xxxx (xx.x) | xxxx/xxxx (xx.x) | x.xx (x.xx, x.xx) | x.xxx | x.xxx |
| Non-sequential devises compared to control** | xxxx/xxxx (xx.x) | xxxx/xxxx (xx.x) | x.xx (x.xx, x.xx) | x.xxx |  |

*Incidence rate of proximal DVT among patients in this subgroup of the IPC group will be compared with Incidence rate of proximal DVT among control group.
